# Supplementary material for: Analysis of factors influencing hookwire dislodgement in CT-guided hookwire localization: a retrospective study using variable importance analysis with a random forest model
Source: PeerJ. 2025 Apr 16;13:e19231. doi: 10.7717/peerj.19231 (PMC12009025; doi:10.7717/peerj.19231)
Supplement: Supplemental Information 4 [file peerj-13-19231-s004.docx]

# 만휘트니 검정 수행 (Perform Mann-Whitney test)

# 데이터 균형 조정 (Balance data using SMOTE)

# balanced_data 데이터프레임에서 dislog 변수를 제외한 나머지 변수 선택 (Select all variables except 'dislog' from balanced_data)

# 결과 확인 (Check results)

###랜덤포레스트 (Random Forest)

# 혼동 행렬 생성 (Create confusion matrix)

# 정확도 계산 (Calculate accuracy)

# 지표들 (Performance metrics)

# 주요 지표 계산 (Calculate key metrics)

# 신뢰구간 계산 (Calculate confidence intervals)

alpha <- 0.05 # 95% 신뢰수준 (Confidence level)

# 민감도 신뢰구간 (Sensitivity confidence interval)

# 특이도 신뢰구간 (Specificity confidence interval)

# 양성예측도 신뢰구간 (Positive Predictive Value confidence interval)

# 음성예측도 신뢰구간 (Negative Predictive Value confidence interval)

# 변수 중요도 추출 (Extract variable importance)

# 변수 중요도 정렬 (Sort variable importance)

# 트리 깊이 계산 함수 (Function to calculate tree depth)

# 루트 노드는 깊이 1 (Root node depth is 1)

# 왼쪽 자식 노드 (Left child node)

# 오른쪽 자식 노드 (Right child node)

# 모든 트리의 깊이 계산 (Calculate depths of all trees)

# 결과 출력 (Print results)
