# Supplementary material for: Analysis of factors influencing hookwire dislodgement in CT-guided hookwire localization: a retrospective study using variable importance analysis with a random forest model
Source: PeerJ. 2025 Apr 16;13:e19231. doi: 10.7717/peerj.19231 (PMC12009025; doi:10.7717/peerj.19231)
Supplement: Supplemental Information 5 [file peerj-13-19231-s005.docx]

Table S1. Clinical and general characteristics of the 240 cases obtained through oversampling using the SMOTE technique

|  | Dislodgement group | Non-dislodgement group | Total |
| --- | --- | --- | --- |
|  | N=105  (15 original cases and 90 synthetic cases) | N=135  (random sampling with replacement from 108 original cases) | N=240 |
| Sex^b^ |  |  |  |
| 1. Male | 46 (43.8%) | 80 (59.3%) | 126 (52.6%) |
| 2. Female | 59 (56.2%) | 55 (40.7%) | 114 (47.5%) |
| Age (years)^a^ | 67.7 ± 10.3 | 66.2 ± 9.3 | 66.9 ± 9.7 |
| Emphysema^b^ |  |  |  |
| 1. None | 59 (56.2%) | 77 (57.0%) | 136 (56.7%) |
| 2. Trace or mild | 46 (43.8%) | 49 (36.3%) | 95 (39.6%) |
| 3. Moderate | 0 (0.0%) | 4 (3.0%) | 4 (1.7%) |
| 4. Confluent | 0 (0.0%) | 5 (3.7%) | 5 (2.1%) |
| 5. Advanced destructive | 0 (0.0%) | 0 (0.0%) | 0 (0.0%) |
| Nodule subtype^b^ |  |  |  |
| 1. Solid | 47 (44.8%) | 86 (63.7%) | 133 (55.4%) |
| 2. PSNs | 54 (51.4%) | 26 (19.3%) | 80 (33.3%) |
| 3. GGNs | 4 (3.8%) | 23 (17.0%) | 27 (11.2%) |
| Nodule size (mm)^a^ | 11.9 ± 5.7 | 10.9 ± 6.1 | 11.4 ± 6.0 |
| Total depth (mm)^a^ | 57.9 ± 11.3 | 59.4 ± 16.9 | 58.7 ± 14.7 |
| Chest wall depth (mm)^a^ | 42.6 ± 9.4 | 40.7 ± 10.2 | 41.5 ± 9.9 |
| Muscle depth (mm)^a^ | 19.4 ± 8.6 | 18.4 ± 9.4 | 18.9 ± 9.0 |
| Distance between nodule to the pleura (mm)^a^ | 15.0 ± 8.9 | 15.9 ± 13.9 | 15.5 ± 12.0 |
| Distance between wire tip to pleura (mm)^a^ | 22.9 ± 11.9 | 31.1 ± 15.4 | 27.5 ± 14.5 |
| Presence of nodule penetration^b^ |  |  |  |
| 1. No | 56 (53.3%) | 76 (56.3%) | 132 (55.0%) |
| 2. Penetrated | 49 (46.7%) | 59 (43.7%) | 108 (45.0%) |
| Procedure time (minutes)^a^ | 19.4 ± 13.3 | 16.2 ± 6.6 | 17.6 ± 10.2 |
| PS interval (minutes)^a^ | 129.1 ± 51.6 | 107.7 ± 49.0 | 117.1 ± 51.2 |
| Pneumothorax^b^ |  |  |  |
| 1. None | 69 (65.7%) | 64 (47.4%) | 133 (55.4%) |
| 2. Yes | 36 (34.3%) | 71 (52.6%) | 107 (44.6%) |
| Hemorrhage^b^ |  |  |  |
| 1. None | 41 (39.0%) | 86 (63.7%) | 127 (52.9%) |
| 2. Yes | 64 (61.0%) | 49 (36.3%) | 113 (47.1%) |
| Presence of multiple localization^b^ |  |  |  |
| 1. No | 76 (72.4%) | 128 (94.8%) | 204 (85.0%) |
| 2. Yes | 29 (27.6%) | 7 (5.2%) | 36 (15.0%) |

Note: PS interval, the interval between the completion of the localization procedure and the initiation of surgery

^a^Data are mean ± standard deviation.

^b^Data are number of patients, with percentages in parentheses.

^*^Result in bold indicates a significant finding.

P-value was calculated with Mann-Whitney test.
