# Supplementary material for: Analysis of factors influencing hookwire dislodgement in CT-guided hookwire localization: a retrospective study using variable importance analysis with a random forest model
Source: PeerJ. 2025 Apr 16;13:e19231. doi: 10.7717/peerj.19231 (PMC12009025; doi:10.7717/peerj.19231)
Supplement: Supplemental Information 6 [file peerj-13-19231-s006.docx]

STROBE Statement—checklist of items that should be included in reports of observational studies

|  | Item No | Recommendation |
| --- | --- | --- |
| **Title and abstract** | 1 | (☑) Indicate the study’s design with a commonly used term in the title or the abstract  : This retrospective study reviewed 123 cases of CT-guided hookwire localization followed by VATS resection. (page 2, line 29) |
|  |  | (☑) Provide in the abstract an informative and balanced summary of what was done and what was found  : Variables analyzed included sex, age, nodule size, emphysema, chest wall/muscle/total depth, distance from the nodule (DNP) or wire tip to the pleura (DWP), procedure time, nodule subtypes, multiple localization, post-procedural hemorrhage, pneumothorax, nodule penetration, and time intervals between completion of procedure to initiation of surgery (PS interval). Variables were compared using chi-square tests or Mann-Whitney tests. A Random Forest model, enhanced with the SMOTE technique for oversampling, was employed to determine the relative importance of each variable. Among the 123 cases, dislodgement occurred in 15. In univariable analysis, only the PS interval was statistically significant (134.1 ± 73.1 vs. 104.1 ± 46.1 minutes in dislodgement or non-dislodgement, *p*=0.031). The Random Forest model identified the top five important variables as the PS interval, DWP, DNP, total depth, and age (page 2, line 30-40). |
| Introduction | | |
| Background/rationale | 2 | ☑Explain the scientific background and rationale for the investigation being reported  : Despite the availability of various techniques for preoperative localization of pulmonary nodules, CT-guided hookwire localization remains one of the oldest and most commonly used methods, with success rate of 93.6-97.6% (11-13). However, hookwire dislodgement is a significant issue with this method, potentially leading to the loss of intraoperative reference for the tumor (14-16). Dislodgement not only poses a challenge during surgery but can also necessitate additional procedures.  Despite the importance of this issue, there is paucity of empirical studies identifying the factors crucial for nodule localization (page 3, line 60-65) |
| Objectives | 3 | ☑State specific objectives, including any prespecified hypotheses  : Despite the importance of this issue, there is paucity of empirical studies identifying the factors crucial for nodule localization. Therefore, this study aims to investigate the risk factors associated with dislodgement of CT-guided hookwire localization (page 3, line 66-68). |
| Methods | | |
| Study design | 4 | ☑Present key elements of study design early in the paper  : We reviewed medical records from January 2012 to May 2024 at one tertiary institution to investigate cases of CT-guided hookwire localization and subsequent VATS resection for lung nodules. We checked where the surgical records documented the success or failure of localization, and cases without documented localization outcomes were excluded from the study. Cases in which localization was performed on two nodules during a single surgery, followed by VATS resection for both nodules, were also included. In such instances, each nodule was considered as a separate case for the purposes of this study. For example, if a single patient underwent localization for two nodules, it was classified and analyzed as two separate cases (Figure 1) (page 4, line 76-84). |
| Setting | 5 | ☑Describe the setting, locations, and relevant dates, including periods of recruitment, exposure, follow-up, and data collection  : We reviewed medical records from January 2012 to May 2024 at one tertiary institution to investigate cases of CT-guided hookwire localization and subsequent VATS resection for lung nodules. We checked where the surgical records documented the success or failure of localization, and cases without documented localization outcomes were excluded from the study. Cases in which localization was performed on two nodules during a single surgery, followed by VATS resection for both nodules, were also included. In such instances, each nodule was considered as a separate case for the purposes of this study. (page 4, line 76-84). |
| Participants | 6 | (☑) *Cohort study*—Give the eligibility criteria, and the sources and methods of selection of participants. Describe methods of follow-up  *Case-control study*—Give the eligibility criteria, and the sources and methods of case ascertainment and control selection. Give the rationale for the choice of cases and controls  *Cross-sectional study*—Give the eligibility criteria, and the sources and methods of selection of participants  : We reviewed medical records from January 2012 to May 2024 at one tertiary institution to investigate cases of CT-guided hookwire localization and subsequent VATS resection for lung nodules. We checked where the surgical records documented the success or failure of localization, and cases without documented localization outcomes were excluded from the study. Cases in which localization was performed on two nodules during a single surgery, followed by VATS resection for both nodules, were also included. In such instances, each nodule was considered as a separate case for the purposes of this study. (page 4, line 76-84).  : The surgeon documented whether the wire localization was well positioned in the operation field. We retrospectively reviewed the operation notes, and cases where the documentation did not clearly state were excluded from the analysis (n=24). The initiation and completion times of the surgery were also recorded to evaluate PS interval (page 6, line 119-122). |
|  |  | (*b*) *Cohort study*—For matched studies, give matching criteria and number of exposed and unexposed  *Case-control study*—For matched studies, give matching criteria and the number of controls per case |
| Variables | 7 | ☑Clearly define all outcomes, exposures, predictors, potential confounders, and effect modifiers. Give diagnostic criteria, if applicable  : The surgeon documented whether the wire localization was well positioned in the operation field. We retrospectively reviewed the operation notes, and cases where the documentation did not clearly state were excluded from the analysis (n=24). The initiation and completion times of the surgery were also recorded to evaluate PS interval (Page 6, line 119-122).  : We reviewed medical records from January 2012 to May 2024 at one tertiary institution to investigate cases of CT-guided hookwire localization and subsequent VATS resection for lung nodules (page 4, line 76-78).  : The maximum diameter of the nodule from skin (total depth), the thickness of the chest wall and muscle layer traversed by the localization hookwire, distance from the wire tip and the pleura, distance from the nodule to the pleura were measured using electronic calipers (millimeters) (Figure 2). Additionally, we checked whether the localization hookwire penetrated the nodule. Post procedural pneumothorax and pulmonary hemorrhage were recorded. Pulmonary hemorrhage was defined as new consolidation or GGNs on post-procedural images and was recorded as presence of hemorrhage, including needle tract hemorrhage less than 2 cm in width (17). The initiation and completion times of the CT-guided localization procedure were recorded. These times were utilized to evaluate the interval between the completion of the localization procedure and the initiation of surgery (PS interval). We also assessed cases performing multiple localizations during a single localization procedure (page 5, line 98-109).  : The radiologist evaluated the nodule subtypes and location of the nodule. The nodule subtype was classified into three categories: pure GGNs, part-solid nodules (PSNs), and solid nodules. Degrees of emphysema (0, none; 1, trace or mild; 2, moderate; 3, confluent; 4, advanced destructive) also recorded (page 5, line 112-116). |
| Data sources/ measurement | 8* | ☑For each variable of interest, give sources of data and details of methods of assessment (measurement). Describe comparability of assessment methods if there is more than one group  : CT images obtained during CT guided localization were reviewed by a thoracic radiologist (J.H.H.) who was blinded whether the case was of dislodgement or not. The maximum diameter of the nodule from skin (total depth), the thickness of the chest wall and muscle layer traversed by the localization hookwire, distance from the wire tip and the pleura, distance from the nodule to the pleura were measured using electronic calipers (millimeters) (page 5, line 97-101).  : Preoperative CT scans were reviewed by a thoracic radiologist (J.Y.K). The radiologist evaluated the nodule subtypes and location of the nodule (page 5, line 112-113).  : The surgeon documented whether the wire localization was well positioned in the operation field. We retrospectively reviewed the operation notes, and cases where the documentation did not clearly state were excluded from the analysis (n=24) (page 6, line 119-121). |
| Bias | 9 | ☑Describe any efforts to address potential sources of bias  : We retrospectively reviewed the operation notes, and cases where the documentation did not clearly state were excluded from the analysis (n=24) (page 6, line 120-121). |
| Study size | 10 | ☑Explain how the study size was arrived at  : A total of 141 patients were identified, of which 24 were excluded due to unclear documentation of localization dislodgement in the surgical records. Six patients underwent two localization procedures simultaneously. A total of 123 procedures were performed on 117 patients (page 8, line 167-170). |
| Quantitative variables | 11 | ☑Explain how quantitative variables were handled in the analyses. If applicable, describe which groupings were chosen and why  : The groups were classified based on the presence or absence of dislodgement. The characteristics of each group were then summarized by presenting the mean and standard deviation. We conducted Mann-Whitney test for comparing continuous variables among both groups, due to the small number of dislodgement cases (n=15) (page 6, line 125-128).  : Due to the significant imbalance in the data distribution, with group sizes of 15 and 108, it was deemed inappropriate to classify the data using a Random Forest model directly. Thus, the Synthetic Minority Over-sampling Technique (SMOTE) method was applied to address this issue. SMOTE addresses data imbalance by synthesizing new samples for the minority class and adjusting the majority class through oversampling and undersampling (or random sampling), respectively (page 6 and 7, line 140-145). |
| Statistical methods | 12 | (☑) Describe all statistical methods, including those used to control for confounding  : The groups were classified based on the presence or absence of dislodgement. The characteristics of each group were then summarized by presenting the mean and standard deviation. We conducted Mann-Whitney test for comparing continuous variables among both groups, due to the small number of dislodgement cases (n=15). Chi-squared test with Yates's correction was performed for comparing categorical variables (page 6, line 125-129).  To identify factors that may influence the occurrence of dislodgement, we employed a Random Forest model. Random Forest is an ensemble learning technique that enhances predictive performance by aggregating multiple decision trees. Each tree is trained on different samples, which increases the model’s robustness. The model is trained by repeatedly sampling a subset of the data with replacement (bootstrapping) to create multiple decision trees. Each tree in the forest is built from a different bootstrap sample, which typically includes about two-thirds of the original data. The remaining one-third of the data, known as out-of-bag (OOB) samples, which are not used in the training of each tree, allow for an assessment of the model's accuracy and stability without the need for separate validation data (18). The number of tree was set to 500, and the number of variables tried at each split was set to 4.  Due to the significant imbalance in the data distribution, with group sizes of 15 and 108, it was deemed inappropriate to classify the data using a Random Forest model directly. Thus, the Synthetic Minority Over-sampling Technique (SMOTE) method was applied to address this issue. SMOTE addresses data imbalance by synthesizing new samples for the minority class and adjusting the majority class through oversampling and undersampling (or random sampling), respectively (page 6 and 7, line 130-145). |
|  |  | (☑) Describe any methods used to examine subgroups and interactions  : The groups were classified based on the presence or absence of dislodgement. The characteristics of each group were then summarized by presenting the mean and standard deviation. We conducted Mann-Whitney test for comparing continuous variables among both groups, due to the small number of dislodgement cases (n=15). Chi-squared test with Yates's correction was performed for comparing categorical variables (page 6, line 125-129). |
|  |  | (☑) Explain how missing data were addressed  : The surgeon documented whether the wire localization was well positioned in the operation field. We retrospectively reviewed the operation notes, and cases where the documentation did not clearly state were excluded from the analysis (n=24). The initiation and completion times of the surgery were also recorded to evaluate PS interval (page 6, line 119-122). |
|  |  | (☑) *Cohort study*—If applicable, explain how loss to follow-up was addressed  *Case-control study*—If applicable, explain how matching of cases and controls was addressed  *Cross-sectional study*—If applicable, describe analytical methods taking account of sampling strategy  : The surgeon documented whether the wire localization was well positioned in the operation field. We retrospectively reviewed the operation notes, and cases where the documentation did not clearly state were excluded from the analysis (n=24). The initiation and completion times of the surgery were also recorded to evaluate PS interval (page 6, line 119-122). |
|  |  | (☑) Describe any sensitivity analyses  : To identify factors that may influence the occurrence of dislodgement, we employed a Random Forest model. Random Forest is an ensemble learning technique that enhances predictive performance by aggregating multiple decision trees. Each tree is trained on different samples, which increases the model’s robustness. The model is trained by repeatedly sampling a subset of the data with replacement (bootstrapping) to create multiple decision trees. Each tree in the forest is built from a different bootstrap sample, which typically includes about two-thirds of the original data. The remaining one-third of the data, known as out-of-bag (OOB) samples, which are not used in the training of each tree, allow for an assessment of the model's accuracy and stability without the need for separate validation data (18). The number of tree was set to 500, and the number of variables tried at each split was set to 4.  Due to the significant imbalance in the data distribution, with group sizes of 15 and 108, it was deemed inappropriate to classify the data using a Random Forest model directly. Thus, the Synthetic Minority Over-sampling Technique (SMOTE) method was applied to address this issue. SMOTE addresses data imbalance by synthesizing new samples for the minority class and adjusting the majority class through oversampling and undersampling (or random sampling), respectively. In SMOTE, oversampling of the minority class (the group with dislodgement) was performed using the K-nearest neighbors (KNN) algorithm. Specifically, KNN was employed to identify the K-nearest neighbors for each minority class data point, and new synthetic data points were generated by creating linear combinations of these neighbors and the existing data points (19). The value of K was set to 5, and the minority class data was increased by 600%. For the majority class (the group without dislodgement), random sampling with replacement was conducted to match 150% of the newly oversampled minority class.  The results of the random forest model were described using OOB (out-of-bag) data, focusing on the sensitivity, specificity, accuracy of the predicted values compared to the actual values, and the P-value (for accuracy > no information rate). Additionally, the model was applied to the original dataset without SMOTE, and the sensitivity, specificity, accuracy, and P-value were also described. The importance of variables, as results of random forest model, were presented using Mean Decrease Accuracy (MDA) and Mean Decrease Gini (MDG). These metrics provide insights into the relative importance of each predictor in the model. MDA measures the importance of each variable by calculating the decrease in model accuracy when the variable is excluded. MDG measures the importance of each variable by calculating the decrease in the Gini impurity when the variable is used in the tree splits (20) (page 6 and 7, line 130-161). |

Continued on next page

| Results | | |
| --- | --- | --- |
| Participants | 13* | (☑) Report numbers of individuals at each stage of study—eg numbers potentially eligible, examined for eligibility, confirmed eligible, included in the study, completing follow-up, and analysed  : A total of 141 patients were identified, of which 24 were excluded due to unclear documentation of localization dislodgement in the surgical records. Six patients underwent two localization procedures simultaneously. A total of 123 procedures were performed on 117 patients (page 8, line 167-170). |
|  |  | (☑) Give reasons for non-participation at each stage  : A total of 141 patients were identified, of which 24 were excluded due to unclear documentation of localization dislodgement in the surgical records (page 8, line 167-168). |
|  |  | (☑) Consider use of a flow diagram  : Figure 1. Case inclusion flow chart (page 16, line 2). |
| Descriptive data | 14* | (☑) Give characteristics of study participants (eg demographic, clinical, social) and information on exposures and potential confounders  : The general characteristics of the participants are presented in Table 1. Among 123 included cases, 15 cases (12.2 %; M:F, 7:8) showed wire dislodgement. The mean age was 67.2 ± 14.0 years in the dislodgement group and 66.0 ± 10.7 years in the non-dislodgement group. Of these dislodged 15 cases, 8 nodules were located in the lower lobes (right lower lobe, 5; left lower lobe, 3), and 7 were in the upper or middle lobes (right upper lobe, 5; right middle lobe, 1; left upper lobe, 1). The mean nodule size was 11.7 mm (range, 5 mm to 28 mm). Seven cases were solid nodules, 7 were PSNs and 1 case was GGN. Among these, 7 nodules were penetrated with the hookwire, while 8 were not. Age, Sex, emphysema degree, nodule subtypes (solid, GGNs or PSNs) and nodule size were not statistically significant differences between the dislodgement and non-dislodgement groups (Table 1) (page 8, line 173-182). |
|  |  | (☑) Indicate number of participants with missing data for each variable of interest  : : A total of 141 patients were identified, of which 24 were excluded due to unclear documentation of localization dislodgement in the surgical records (page 8, line 167-168). |
|  |  | (c) *Cohort study*—Summarise follow-up time (eg, average and total amount) |
| Outcome data | 15* | ☑*Cohort study*—Report numbers of outcome events or summary measures over time |
|  |  | *Case-control study—*Report numbers in each exposure category, or summary measures of exposure |
|  |  | *Cross-sectional study—*Report numbers of outcome events or summary measures  : The general characteristics of the participants are presented in Table 1. Among 123 included cases, 15 cases (12.2 %; M:F, 7:8) showed wire dislodgement. The mean age was 67.2 ± 14.0 years in the dislodgement group and 66.0 ± 10.7 years in the non-dislodgement group. Of these dislodged 15 cases, 8 nodules were located in the lower lobes (right lower lobe, 5; left lower lobe, 3), and 7 were in the upper or middle lobes (right upper lobe, 5; right middle lobe, 1; left upper lobe, 1). The mean nodule size was 11.7 mm (range, 5 mm to 28 mm). Seven cases were solid nodules, 7 were PSNs and 1 case was GGN. Among these, 7 nodules were penetrated with the hookwire, while 8 were not. Age, Sex, emphysema degree, nodule subtypes (solid, GGNs or PSNs) and nodule size were not statistically significant differences between the dislodgement and non-dislodgement groups (Table 1). Despite the dislodgement, resections of all 15 cases were successfully performed, although 2 cases were converted to lobectomy instead of wedge resection. Both cases were confirmed as lung adenocarcinoma, and lobectomy was appropriated treatment for them.  During the localization procedure, parenchymal hemorrhage was observed in 53 cases (43%). Pneumothorax occurred in 61 cases (49%). In the dislodgement group, 5 cases (33.3%) of pneumothorax and 9 cases (60.0%) of parenchymal hemorrhage was occurred. In the non-dislodgement group, 56 cases (51.9%) of pneumothorax and 44 cases (40.7%) of parenchymal hemorrhage was occurred. No statistical significant difference were observed in both groups according to post procedural complications. None of the patients required specific intervention associated for post-procedural complications (page 8 and 9, line 173-192). |
| Main results | 16 | (☑) Give unadjusted estimates and, if applicable, confounder-adjusted estimates and their precision (eg, 95% confidence interval). Make clear which confounders were adjusted for and why they were included  : The error rate performed on OOB data was 5.83%. Based on the SMOTE-performed sample, out of 105 cases (including synthetic cases) where dislodgement occurred, 96 were predicted to have dislodgement, resulting in a sensitivity of 91.4%. Among the 135 cases without dislodgement (randomly resampled), 130 were predicted to be free of dislodgement, yielding a specificity of 96.3%. The classification accuracy was 0.94 (95% CI 0.90-0.98), and the P-value (for accuracy > no information rate) was <0.0001, indicating significance.  Considering the model was built with synthetic data, another validation was performed on the original dataset (15 dislodgement cases and 108 non-dislodgement cases). Of the cases predicted to be positive (group where dislodgement occurred), 22 cases were identified, with 15 being actual cases of dislodgement and 7 being false positives. In the group predicted to be negative (group where dislodgement did not occur), 101 cases were identified, with no false negatives.The classification accuracy was 0.94 (95% CI 0.89-0.98), and the P-value (for accuracy > no information rate) was 0.01, indicating significance. Assuming dislodgement cases as positive, the model showed a sensitivity of 1.0, specificity of 0.93 (page 10, line 217-230). |
|  |  | (☑) Report category boundaries when continuous variables were categorized  : The PS interval was significantly different between dislodgement and non-dislodgement group, with 134.1 ± 73.1 minutes in the dislodgement group and 104.1 ± 46.1 minutes in the non-dislodgement group (*p*=0.031). However, total depth (58.4 ± 13.6 mm and 60.2 ± 19.2 mm, respectively), distance from the nodule to the pleura (16.3 ± 11.2 mm and 16.1 ± 14.3 mm, respectively), distance from the wire tip to the pleura (24.6 ± 15.5 mm and 31.4 ± 14.0 mm, respectively), procedure time (19.3 ± 17.5 minutes and 15.3 ± 7.1 minutes, respectively), chest wall depth (41.7 ± 12.0 mm and 41.1 ± 11.9 mm, respectively), muscle depth (18.9 ± 11.2 mm and 19.0 ± 11.0 mm, respectively) were not significantly different between two groups (Table 1) (page 9, lne 193-203) |
|  |  | (*c*) If relevant, consider translating estimates of relative risk into absolute risk for a meaningful time period |
| Other analyses | 17 | ☑Report other analyses done—eg analyses of subgroups and interactions, and sensitivity analyses  : The PS interval was significantly different between dislodgement and non-dislodgement group, with 134.1 ± 73.1 minutes in the dislodgement group and 104.1 ± 46.1 minutes in the non-dislodgement group (*p*=0.031) (page 9, line 193-195).  : Considering the model was built with synthetic data, another validation was performed on the original dataset (15 dislodgement cases and 108 non-dislodgement cases). Of the cases predicted to be positive (group where dislodgement occurred), 22 cases were identified, with 15 being actual cases of dislodgement and 7 being false positives. In the group predicted to be negative (group where dislodgement did not occur), 101 cases were identified, with no false negatives.The classification accuracy was 0.94 (95% CI 0.89-0.98), and the P-value (for accuracy > no information rate) was 0.01, indicating significance (page 10, line 223-229). |
| Discussion | | |
| Key results | 18 | ☑Summarise key results with reference to study objectives  : This study identified factors influencing the dislodgement of CT-guided hookwire localization. Univariate analysis showed that PS interval was the only significant factor for dislodgement. We utilized various multivariate analysis and machine learning techniques such as SMOTE and random forest for deeper analysis. In the random forest model, five factors—distance from the nodule to the pleura, distance from the wire tip to the pleura, total depth, and age—showed relatively high priority in predicting dislodgement. Factors such as the occurrence of pneumothorax during the procedure, presence of nodule penetration, and emphysema demonstrated the lowest importance in classification (page 10 and 11, line 233-240). |
| Limitations | 19 | ☑Discuss limitations of the study, taking into account sources of potential bias or imprecision. Discuss both direction and magnitude of any potential bias  : However, this study has limitations. First, as a cross-sectional study, it cannot establish causality. Second, the relatively small number of cases, especially those with dislodgement, prevented us from performing validation through methods such as train-test set split or k-fold validation. Third, this study is the retrospective study and relied on surgical records for data on dislodgement. To mitigate this, we meticulously reviewed the surgical records and excluded cases with unclear documentation of localization success. Fourth, the measured distance were measured based on axial CT rather than a 3D analysis, potentially leading to minor discrepancies. However, since most CT-guided localization procedures are performed axially, the difference is likely negligible. Fifth, the study was conducted at a single hospital, making it difficult to generalize the results to other hospitals and healthcare systems, and we did not consider operator factors like experience and expertise (page 12, line 268-278). |
| Interpretation | 20 | ☑Give a cautious overall interpretation of results considering objectives, limitations, multiplicity of analyses, results from similar studies, and other relevant evidence  : This study identified factors influencing the dislodgement of CT-guided hookwire localization. Univariate analysis showed that PS interval was the only significant factor for dislodgement. We utilized various multivariate analysis and machine learning techniques such as SMOTE and random forest for deeper analysis. In the random forest model, five factors—distance from the nodule to the pleura, distance from the wire tip to the pleura, total depth, and age—showed relatively high priority in predicting dislodgement. Factors such as the occurrence of pneumothorax during the procedure, presence of nodule penetration, and emphysema demonstrated the lowest importance in classification.  Previous studies assessing successful CT-guided hookwire localization have provided similar insights. Earlier literatures have identified the distance from the wire tip to the pleura as the most important factor for successful CT-guided nodule localization (14, 21).In other study, one out of 17 cases showed dislodgement where the interval between the procedure and surgery was delayed by six hours (22). Our study not only considered the previously noted crucial factor, the distance from the wire tip to the pleura, but also incorporated the time interval between the procedure and surgery. By analyzing these factors, we found that the time interval is an even more crucial factor influencing dislodgement. This suggests that, besides the technical skills of radiologists and thoracic surgeons and patient-specific factors, timely surgery after localization is crucial for successful surgery (page 10 and 11, line 233-250).  In conclusion, this study identified the PS interval as the most critical factor in hookwire dislodgement, along with distance from the nodule or wire tip to the pleura, total depth, and age. These findings highlight the importance of timely surgery post-localization to minimize the risk of dislodgement (page 12 and 13, line 280-283). |
| Generalisability | 21 | ☑Discuss the generalisability (external validity) of the study results  : Fifth, the study was conducted at a single hospital, making it difficult to generalize the results to other hospitals and healthcare systems, and we did not consider operator factors like experience and expertise (page 12, line 276-278). |
| Other information | | |
| Funding | 22 | ☑Give the source of funding and the role of the funders for the present study and, if applicable, for the original study on which the present article is based  : I have stated the following on the PeerJ submission site: 'This work was supported by the Dongguk University, College of Medicine Research Fund of 2024. All authors have no conflicts of interest to declare |

*Give information separately for cases and controls in case-control studies and, if applicable, for exposed and unexposed groups in cohort and cross-sectional studies.

**Note:** An Explanation and Elaboration article discusses each checklist item and gives methodological background and published examples of transparent reporting. The STROBE checklist is best used in conjunction with this article (freely available on the Web sites of PLoS Medicine at http://www.plosmedicine.org/, Annals of Internal Medicine at http://www.annals.org/, and Epidemiology at http://www.epidem.com/). Information on the STROBE Initiative is available at www.strobe-statement.org.
